# Association of *IFNL3* rs12979860 and rs8099917 with Biochemical Predictors of Interferon Responsiveness in Chronic Hepatitis C Virus Infection

**DOI:** 10.1371/journal.pone.0077530

**Published:** 2013-10-29

**Authors:** Janett Fischer, Stephan Böhm, Tobias Müller, Heiko Witt, Christoph Sarrazin, Simone Susser, Pascal Migaud, Eckart Schott, Graeme Stewart, Annika Brodzinski, Balazs Fülöp, Florian van Bömmel, Jacob George, Thomas Berg

**Affiliations:** 1 Universitätsklinikum Leipzig, Sektion Hepatologie, Klinik und Poliklinik für Gastroenterologie und Rheumatologie, Leipzig, Germany; 2 Technische Universität München (TUM), Kinderklinik Schwabing und Else Kröner-Fresenius-Zentrum (EKFZ) Munich, Germany; 3 J. W. Goethe-University Hospital, Medizinische Klinik 1, Frankfurt, Germany; 4 University Hospital Charité, Campus Virchow-Klinikum, Department of Gastroenterology and Hepatology, Berlin, Germany; 5 Institute of Immunology and Allergy Research, Westmead Hospital and Westmead Millennium Institute, University of Sydney, Australia; 6 Storr Liver Unit, Westmead Hospital and Westmead Millennium Institute, University of Sydney, Australia; University of Sydney, Australia

## Abstract

**Background & Aims:**

Genetic variations near the interferon lambda 3 gene (*IFNL3, IL28B*) are the most powerful predictors for sustained virologic response (SVR) in patients with chronic hepatitis C virus (HCV) infection, compared to other biochemical or histological baseline parameters. We evaluated whether the interplay of both *IFNL3* polymorphisms rs12979860 and rs8099917 together with non-genetic clinical factors contributes to the predictive role of these genetic variants.

**Methods:**

The cohort comprised 1,402 patients of European descent with chronic HCV type 1 infection. 1,298 patients received interferon-based antiviral therapy, and 719 (55%) achieved SVR. The *IFNL3* polymorphisms were genotyped by polymerase chain reaction and melting curve analysis.

**Results:**

A significant correlation was found between the *IFNL3* polymorphisms and biochemical as well as virologic predictors of treatment outcome such as ALT, GGT, cholesterol, and HCV RNA levels. In multivariate regression analysis, *IFLN3* SNPs, HCV RNA levels, and the GGT/ALT ratio were independent predictors of SVR. Dependent on the GGT/ALT ratio and on the HCV RNA concentration, significant variations in the likelihood for achieving SVR were observed in both, carriers of the responder as well as non-responder alleles.

**Conclusions:**

Our data support a clear association between *IFNL3* genotypes and baseline parameters known to impact interferon responsiveness. Improved treatment outcome prediction was achieved when these predictors were considered in combination with the *IFNL3* genotype.

## Introduction

Hepatitis C virus (HCV) infection is one of the world’s dominant cause for developing severe liver disease that can progress to cirrhosis (20–30%) and hepatocellular carcinoma (4%) [1]. A link between certain disease characteristics and the natural course of infection and treatment outcome has been demonstrated in many studies. In these reports, baseline predictors, viral factors, host determinants and on-treatment factors, have been shown to influence disease progression and treatment response [2]–[6].

In previous reports, the gamma-glutamylaminotransferase (GGT) level was identified as a significant predictor of virologic response in patients with advanced liver disease [5], [7], [8]. Other studies demonstrated a strong association of alanine aminotransferase (ALT) activity with treatment outcome [9]. We have reported that an inverse correlation between ALT and GGT exists, where increased levels of GGT together with ALT flares are less predictive for non-response than increased GGT levels without significantly increased ALT levels [5]. Moreover, some studies reported on interactions between cholesterol metabolism and interferon responsiveness; low levels of low density lipid (LDL) cholesterol were associated with a higher chance of HCV clearance [10], [11]. The mechanisms by which these factors influence interferon responsiveness remain to be determined.

Several independent genome-wide associated studies (GWAS) have established strong associations of genetic polymorphisms near the *IFNL3* (interferon lambda 3, IL28B) gene locus, especially rs12979860 and rs8099917, with treatment outcome [12]–[16] and spontaneous viral clearance [17], [18], [19]. The preferred variants rs12979860CC and rs8099917TT are significantly associated with a sustained virologic response (SVR) in HCV genotype 1-infected patients treated with pegylated interferon (PegIFN) alpha and ribavirin [6], [20], [21]. Additionally, some reports provided evidence that *IFNL3* polymorphisms influence the efficacy of different protease-based triple regimens [22], [23].

The functional link and molecular pathways between the *IFNL3* polymorphisms and treatment-induced HCV clearance remain far from clear. Nonetheless, it has been shown that the intrahepatic expression profiles of interferon stimulated genes (ISG) vary according to different *IFNL3* rs12979860 and rs8099917 genotypes [16], [24]. Patients carrying the favorable *IFNL3* genotypes showed degreased expression levels of genes promoting antiviral state and an increased expression of ISG suppressors. Low pre-treatment ISG levels have been found to be associated with a successful IFN-based therapy whereas patients having high ISG levels poorly respond to interferon, because the genes are already activated at an intermediate level and are refractory to further induction by enthetic IFN [25]–[28].

However, *IFNL3* genotypes may also have an impact on biochemical parameters such as GGT, ALT and LDL levels, and thereby provide a link to the well known predictive impact of baseline predictors of treatment outcome [9]. Indeed, Amanzada et al. [29] argued that the GGT/ALT ratio may enhance the SVR predictability of the *IFNL3* rs12979860CC genotype.

Recently, a new polymorphism (ss469415590, ΔG/TT) was identified between the *IFNL2* and *IFNL3* genes which creates or disrupts an open reading frame in a new gene designated interferon lambda 4 (*IFNL4*) [30]–[32]. It was suggested that the presence of the functional interferon lambda 4 is associated with impaired HCV clearance and treatment response. However, because of its high linkage to the *IFNL3* rs12969860 SNP in patients of European descent *IFNL4* provides comparable information.

To further increase our understanding of the relationship between *IFNL3* genotype and treatment outcome, we undertook a large multicentre, retrospective association study of the *IFNL3* polymorphisms rs12979860 and rs8099917 with baseline parameters and disease characteristics in HCV infection and defined their relative importance.

## Patients and Methods

### Ethics Statement

The study was approved by the Ethics Committees of Medical Research of the University of Leipzig and of Berlin and the Human Research Ethics committees of Sydney West Area Health Service and the University of Sydney in accordance with the 1975 Declaration of Helsinki. The INDIV-1 and INDIV-2 studies were enrolled at 19 and 20 centers in Germany, respectively. Independent ethics committee approval had been received at each center according to the Declaration of Helsinki and the International Conference on Harmonization/Committee for Proprietary Medicinal Products “Good Clinical Practice” guidelines. All patients provided written informed consent.

### Patients

The evaluation cohort (EC) comprised 1000 patients of European descent with chronic HCV type 1 infection from Germany (882) and Australia (118). Parts of the cohort were included in the original GWAS [13] and in the response-guided individualized tailored treatment regimen of the INDIV-2 study [33]. The median age was 46 years and 549 (55%) patients were male. Baseline parameters included aspartate aminotransferase (AST), alanine aminotransferase (ALT), alkaline phosphatase (AP), gamma-glutamylaminotransferase (GGT), cholesterol, triglycerides, bilirubin and platelets. The AST-platelet-ratio (APRI score) was calculated using the formula; [APRI score  =  (AST level (ULN)/platelet counts (10^9^/L))*100] [34]. Liver biopsy was performed in 848 patients and analyzed by local pathologists at the clinics. Histological inflammatory activity and fibrosis stages were scored according to the Desmet-Scheuer [35] and METAVIR [36] score. Liver steatosis was evaluated by ultrasonography and by calculating the percentage of lipid droplets containing hepatocytes of total number of hepatocytes. The characteristics of the study cohort are shown in Table 1.

**Table 1:** Patients’ characteristics of the evaluation and replication cohort.

Parameter	Evaluation cohort (EC)		Replication cohort (RC)	
**Number of patients**		1000		402
**Age (years)**		46 (39, 54)^a^		47 (40, 56)^a^
**Gender (male/female)**		55%/45%		51%/49%
**ALT (IU/L)**		61 (38, 97)^a^		72 (45, 111)^a^
**AST (IU/L)**		43 (29, 65)^a^		49 (33, 72)^a^
**GGT (IU/L)**		47 (24, 87)^a^		54 (28, 101)^a^
**GGT/ALT ratio**		0.8 (0.4, 1.4)^a^		0.7 (0.4, 1.3)^a^
**AST/ALT ratio**		0.7 (0.6, 0.9)^a^		0.7 (0.6, 0.9)^a^
**APRI score**	n = 492	0.8 (0.5, 1.7)^a^	n = 263	0.2 (0.4, 1.0)^a^
**AP (IU/L)**	n = 505	91 (70, 119)^a^	n = 279	96 (73, 132)^a^
**Cholesterol (mg/dL)**	n = 632	170 (147, 196)^a^	n = 335	177 (156, 200)^a^
**Triglycerides (mg/dL)**	n = 612	93 (67, 133)^a^	n = 320	96 (74, 143)^a^
**Bilirubin (mg/dL)**	n = 665	0.7 (0.4, 0.9)^a^	n = 309	0.6 (0.3, 0.8)^a^
**Platelets (10^9^/L)**	n = 492	203 (157, 249)^a^	n = 263	222 (176, 265)^a^
**HCV RNA Log_10_ (IU/mL)**	n = 923	5.8 (5.3, 6.2)^a^	n = 379	5.8 (5.3, 6.2)^a^
**Steatosis**				
**None/<33%/33%–65%/≤100%**	n = 401	16%/64%/17%/3%	n = 256	65%/26%/6%/3%
**Fibrosis stage**				
**F0/F1/F2/F3/F4**	n = 848	16%/38%/26%/13%/7%	n = 370	19%/35%/24%/12%/10%
**Cirrhosis**	n = 848	9%	n = 370	11%
**Histological activity**				
**A0/A1/A2/A3**	n = 792	5%/42%44%/9%	n = 327	8%/46%/40%/6%
**Treatment outcome**				
**SVR/NR/Relapse**	n = 955	46%/35%/19%	n = 343	42%/41%/17%

a median plus interquartile range (25^th^, 75^th^ percentile), SVR: sustained virologic response, NR: non-response, IU: international units.

Chronic HCV infection was diagnosed by a positive anti-HCV test in routine diagnostic and by presence of HCV RNA in serum for more than 6 months. HCV RNA concentration was determined by qualitative (TMA) and quantitative (bDNA, Cobas® Amplicor Analyzer and high sensitive realtime PCR) assays. 955 patients were treated with interferon-based therapy consisting of pegylated interferon (IFN) and ribavirin. They received the recommended doses and were adherent. Treatment duration ranged from 24 to 72 weeks depending on the individual on-treatment response. The standard treatment duration of 48 weeks was applied to 659 (69%) patients. An individualized treatment with 49–72 weeks was applied to 105 patients (11%) that were part of the INDIV-2 study [33]. 191 (20%) patients of the INDIV-2 study had a treatment duration of 24–43 weeks. 435 (46%) patients had sustained virological response (SVR), determined as undetectable HCV RNA levels 6 months after completion of therapy. All other patients were classified as patients with non-sustained virological response (non-SVR). The non-SVR cohort included patients with either non-response (N = 336) or relapse (n = 184). An independent replication cohort (RC) of 402 HCV type 1-infected patients, including 264 Caucasian patients of the INDIV-1 study [6] was analyzed (Table 1). 343 patients were treated with dual interferon-based therapy. 239 (70%) patients of the replication cohort had standard treatment duration of 48 week. An individualized treatment with 24–45 weeks was applied to 104 (30%) patients. 143 (42%) patients had SVR.

### Methods

The DNA samples of all patients were analyzed for the *IFNL3* SNPs rs12979860 and rs8099917 SNPs although data for some parts of the cohort were already available by GWAS [13]. For genotyping we performed real-time polymerase chain reaction and melting curve analysis in a Light Cycler 480 System (Roche, Mannheim, Germany) as described elsewhere [37].

### Statistical Analysis

Statistical analysis was performed with SPSS 20.0 (SPSS, Chicago, Illinois, USA). The significance of associations between dichotomous data was assessed by Pearson’s χ^2^ test and Fischer’s exact test. Continuous data were explored by Mann-Whitney U-tests. The predictive value of biochemical parameters was analyzed for its receiver operating characteristics (ROC). The area under the ROC (AUROC) curve with values close to 1.0 indicates high diagnostic accuracy. The most accurate cut-off value was calculated by the Youden index [38]. Simple and stepwise multiple regression analyses were performed to determine factors associated with SVR. All tests were two-sided and p-values less than 0.05 were considered statistically significant. The odds ratio (OR) and the 95% confidence interval (CI) were calculated.

## Results

### Genotype Distribution

In the evaluation cohort (EC) of 1000 patients, the overall genotype distribution of rs12979860 CC, CT, and TT was 31%, 53%, and 16%, and the distribution of rs8099917 TT, TG and GG was 50%, 43% and 7%, respectively. The distribution of rs12979860 and rs8099917 in the replication cohort (RC) was similar; 29% CC, 53% CT and 18% TT for rs12979860 and 51% TT, 42% TG and 7% for rs8099917 (Table 2).

**Table 2:** Genotype frequencies and sustained virologic response (SVR) rates of *IFNL3* rs12979860 and rs8099917 SNPs in the evaluation and replication cohort.

		Evaluation cohort		Replication cohort	
Genotype		Frequency (%)	SVR (%)	Frequency (%)	SVR (%)
**rs12979860**	**CC**	31	65	29	57
	**CT**	53	38	53	35
	**TT**	16	31	18	37
**rs8099917**	**TT**	50	58	51	51
	**TG**	43	33	42	30
	**GG**	7	31	7	39

### Baseline Factors Associated with Variants of rs12979860 and rs8099917

**Figure 1:**
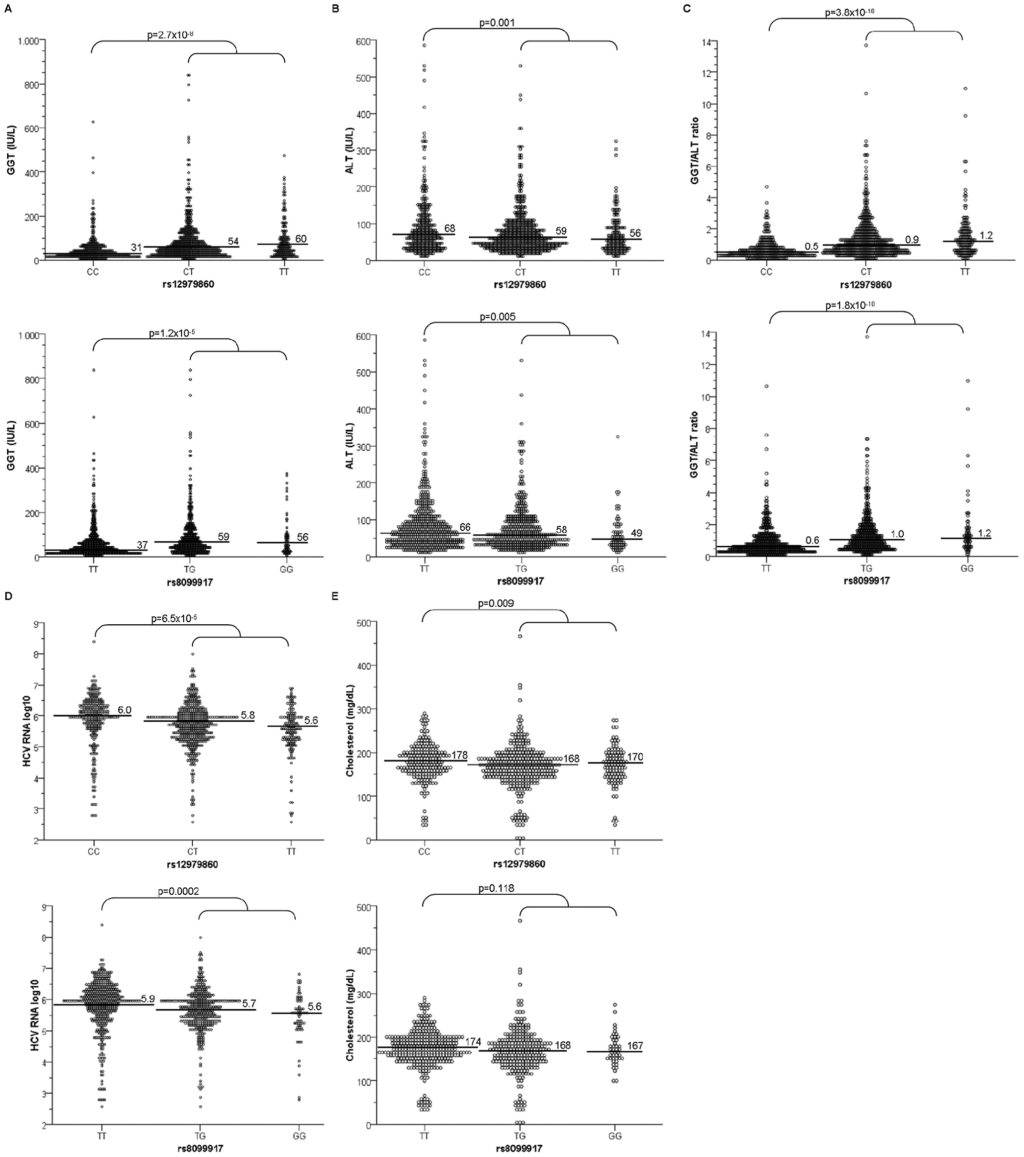
Association of *IFNL3* variants with baseline predictors. Association of the *IFNL3* rs12979860 and rs8099917 genotypes with the levels of (**A**) GGT (IU/mL), (**B**) ALT (IU/mL), (**C**) GGT/ALT ratio, (**D**) pretreatment HCV RNA log_10_ (IU/mL) and (**E**) cholesterol (mg/dL) concentration in the evaluation cohort (EC). Horizontal bars represent the median. Mann-Whitney U-test was used to compare the baseline parameter.

In univariate analyses of both cohorts, carriers of the homozygote rs12979860CC genotype showed significantly lower GGT levels, lower GGT/ALT ratio, higher HCV RNA and ALT levels and less steatosis (EC: p = 0.022; RC: p = 0.05) compared to carriers of at least one non-responder T-allele. The rs8099917TT responder genotype showed a weaker correlation with, low GGT levels, low GGT/ALT ratios and high HCV RNA and ALT levels. In the evaluation cohort, there was also an association of rs12979860CC with elevated cholesterol concentrations (p = 0.009). Figure 1 shows the associations of rs12979860 and rs8099917 with baseline parameters in the EC (RC in Figure S1).

Multiple regression analysis yielded an association of rs12979860CC with low GGT/ALT ratios (EC: OR = 0.12 [0.05–0.30] p = 5.7×10^−6^; RC: OR = 0.27 [0.13–0.54] p = 0.0002), high HCV RNA levels (EC: OR = 2.40 [1.41–4.08] p = 0.001; RC: OR = 2.20 [1.27–3.78] p = 0.005) and high cholesterol concentration in the evaluation cohort (OR = 1.01 [1.00–1.02] p = 0.005). The rs8099917TT was associated with a low GGT/ALT ratio (EC: OR = 0.78 [0.64–0.95] p = 0.012; RC: OR = 0.63 [0.49–0.82] p = 0.0005) and high HCV RNA levels (EC: OR = 1.4 [1.08–1.89] p = 0.012; RC: OR = 1.5 [1.05–2.15] p = 0.026).

### Association between *IFNL3* Variants, Baseline Factors and Treatment Response

Within the group of 955 patients treated with pegIFN and ribavirin, 435 (46%) patients exhibited a SVR, 184 (19%) showed a relapse, and 336 (35%) non-response. The SVR rates of the EC were 65%, 38% and 31% for rs12979860 CC, CT and TT, and 58%, 33% and 31% for rs8099917 TT, TG and GG, respectively. After combination of rs12979860 and rs8099917, the highest SVR rates of 66% were for the combined rs12979860CC/rs8099917TT genotype followed by 49% for rs12979860CT/rs8099917TT and 32% for rs12979860CT/rs8099917TG. Similar results were observed in the RC (Table 2).

In both cohorts, univariate regression analyses revealed that SVR was significantly associated with rs12979860CC, rs8099917TT, low values of GGT and AP, low APRI scores and low HCV RNA levels but high cholesterol concentrations and platelet counts. Responder patients had longer treatment duration, were younger and had less steatosis, fibrosis and cirrhosis compared to the non-responder population. Additionally, lower ratios of AST/ALT and GGT/ALT and female gender were associated with treatment response. In stepwise multivariate regression analyses, low GGT/ALT ratio, and low HCV RNA concentration as well as rs12979860CC were independent predictors of SVR. The rs8099917TT also significantly correlated with SVR (Table 3).

**Table 3:** Univariate and multivariate analyses of factors predictive for sustained virologic response (SVR).

	Evaluation cohort				Replication cohort			
Parameter	Univariate Analysis		Multivariate Analysis		Univariate Analysis		Multivariate Analysis	
	OR [95% CI]	P	OR [95% CI]	P	OR [95% CI]	P	OR [95% CI]	P
**Age (years)**	0.96 [0.94–0.97]	7.7×10^−13^			0.93 [0.91–0.95]	7.5×10^−11^		
**HCV RNA Log_10_ (IU/mL)**	0.61 [0.50–0.74]	3.4×10^−7^	0.61 [0.39–0.95]	0.028	0.57 (0.42–0.78]	0.001	0.43 [0.22–0.84]	0.001
**GGT (IU/mL)**	0.99 [0.98–0.99]	1.4×10^−14^			0.99 [0.99–1.00]	0.0002		
**GGT/ALT ratio**	0.45 [0.38–0.55]	7.2×10^−16^	0.45 [0.24–0.82]	0.010	0.52 [0.38–0.71]	4.5×10^−5^	0.60 [0.40–0.693]	0.021
**AST/ALT ratio**	0.39 [0.29–0.70]	0.0004			0.42 [0.19–0.91]	0.027		
**AP (IU/mL)**	0.99 [0.99–1.00]	0.003			0.99 [0.99–1.00]	0.001		
**Platelets (10^9^/L)**	1.01 [1.00–1.01]	1.6×10^−5^			1.01 [1.00–1.02]	2.9×10^−6^		
**Cholesterol (mg/dL)**	1.01 [1.00–1.01]	0.009			1.01 [1.00–1.02]	0.018		
**APRI score**	0.87 [0.77–0.97]	0.014			0.71 [0.53–0.94]	0.016		
**Steatosis**	0.50 [0.29–0.87]	0.013			0.38 [0.21–0.68]	0.001		
**Fibrosis stage F3–F4**	0.49 [0.37–2.70]	8.2×10^−7^			2.79 [1.68–4.63]	6.9×10^−5^		
**Cirrhosis**	0.27 [0.15–0.50]	3.6×10^−5^			0.10 [0.02–0.44]	0.002		
**Female sex**	1.32 [1.02–1.70]	0.036			1.69 [1.10–2.61]	0.017		
**Treatment duration**	1.03 [1.02–1.04]	1.2×10^−7^			1.04 [1.01–1.06]	0.005		
**rs12979860CC**	3.28 [2.46–4.37]	4.0×10^−17^	4.22 [1.76–10.15]	0.001	2.42 [1.51–3.89]	0.0003	4.57 [1.71–12.22]	0.002
**rs8099917TT**	2.84 [2.18–3.70]	9.0×10^−14^	2.36 [1.514–4.91]	0.021	2.27 [1.46–3.53]	0.0003	2.33 [1.07–5.08]	0.013

OR: odds ratio, CI: confidence interval, P = p-value, IU: international units.

Besides the *IFNL3* rs12979860CC and rs8099917TT genotypes, sensitivity and specificity analysis identified the GGT/ALT ratio (cut-off value 0.70) and the baseline HCV RNA concentration (cut-off value 5.8log_10_) as accurate predictors for SVR (Table S1).

**Figure 2:**
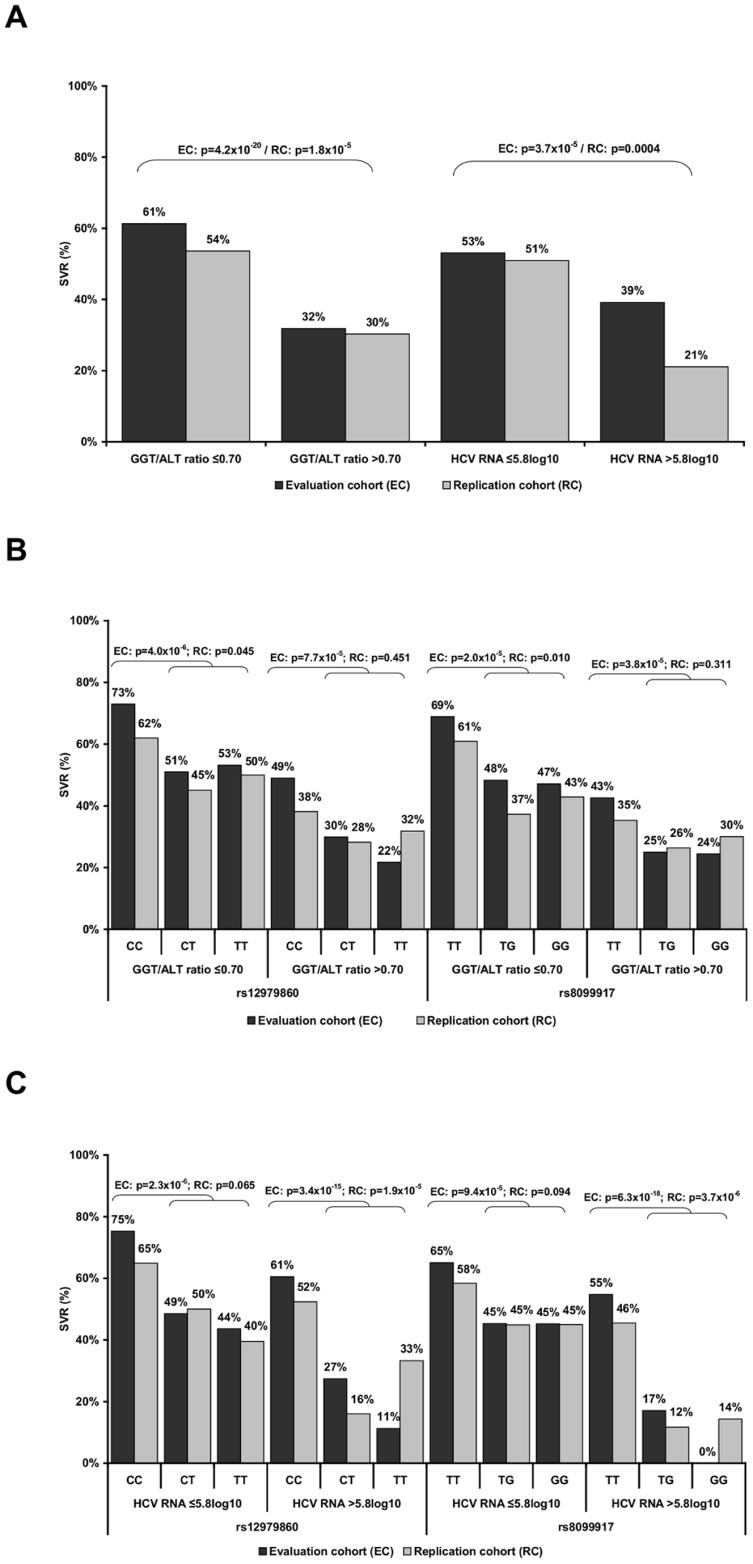
Impact of *IFNL3* variants and baseline predictors on sustained virologic response (SVR). SVR rates in the evaluation (EC) and replication cohort (RC) with regard to (**A**) GGT/ALT ratio and HCV RNA concentration according to *IFNL3* rs12979860 and rs8099917 genotypes in combination and with (**B**) GGT/ALT ratio cutoff value of 0.70, (**C**) HCV RNA concentration cut-off value of 5.8log_10_ (IU/mL). Pearson’s χ^2^ test and Fischer’s exact test were used to compare the SVR rates.

In the evaluation cohort, the SVR rate was 61% when GGT/ALT ratio was ≤0.70 and 32% above cut-off and 53% when HCV RNA concentration was ≤5.8log_10_ and 39% in patients with HCV RNA levels above this cut-off (Fig. 2.A). Best performance was observed for the combination of *IFNL3* with the GGT/ALT ratio. Within the group of patients carrying the rs12979860CC, the SVR increased from 65% to 73% when GGT/ALT ratio was ≤0.70 (p = 0.001). Carriers of the rs8099917TT genotype also had an increase from 58% to 69% when the GGT/ALT ratio was ≤0.70 (p = 0.003). When the GGT/ALT ratio was >0.70, the SVR rates of both homozygous responder variants were significantly reduced (rs12979860CC. p = 0.005; rs8099917TT: p = 0.0004). When HCV RNA levels were >5.8log_10,_ the SVR rates degreased in patients carrying the heterozygous or homozygous non-responder T- and G–alleles of rs12979860 (p = 0.0004) and rs8099917 (p = 7.2×10^−6^), respectively, (Fig. 2.B–C).

**Figure 3:**
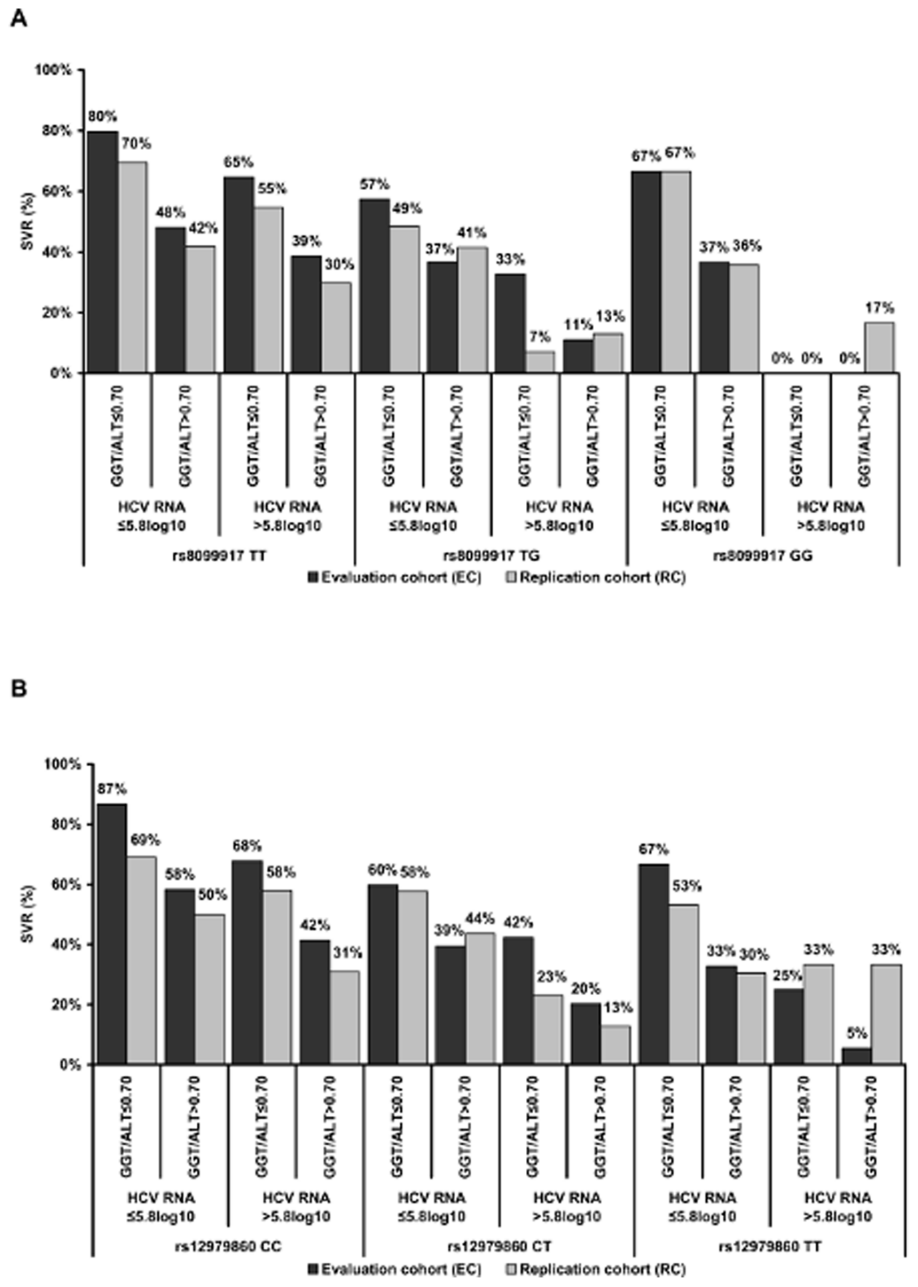
Combined determination of *IFNL3* variants, GGT/ALT ratio and HCV RNA levels improved sustained virologic response (SVR) rates. SVR rates in the evaluation (EC) and replication cohort (RC) according to *IFNL3* (**A**) rs12979860 and (**B**) rs8099917 genotypes after adjustment for the GGT/ALT ratio (cut-off value 0.70) and HCV RNA concentration (cut-off value 5.8log_10_).

The adjustment for the GGT/ALT ratio (cut-off value 0.70) and the HCV RNA concentration (cut-off value 5.8log_10_) further improved the SVR rates according to the *IFNL3* genotypes. Highest SVR rates were observed in patients carrying the rs12979860CC (87%) or 8099917TT (80%) variants and having GGT/ALT ratios and HCV RNA levels below the cut-off values. In patients carrying at least one T-and G-alleles, the SVR rates were increased when both the GGT/ALT ratio and HCV RNA concentration were low. However, independent of the *IFNL3* genotypes, the presence of high GGT/ALT ratios correlated with lower SVR rates although the HCV RNA concentration was ≤5.8log_10_ (Fig. 3). Similar results were observed in the RC.

## Discussion

There is growing evidence that genetic variations near the interferon lambda 3 gene (*IFNL3; IL28B*) are the most powerful predictors for sustained virologic response (SVR) in patients with chronic hepatitis C virus (HCV) infection compared to biochemical or histological parameters [6], [12], [13], [15], [20], [21]. The mechanisms by which these parameters influence interferon (IFN) responsiveness remain obscure. In this study we evaluated whether the interplay of both *IFNL3* polymorphisms together with other non-genetic clinical factors such as the level of inflammatory activity, the ALT and GGT levels and metabolic factors such as the serum cholesterol concentration and the occurrence of steatosis is one reason for the outstanding predictive role of *IFNL3* genetic variants.

In the cohort of 1,402 HCV type 1-infected patients of European descent we indentified that besides the *IFNL3* genotypes, pre-treatment HCV RNA levels, cholesterol concentration as well as levels of GGT and ALT were important baseline predictors of IFN responsiveness. This is in agreement with previous studies [5], [7], [8], [10]. However, the intriguing observation from the present study is the close association of baseline parameters with *IFNL3* genotype.

We proved that patients having low baseline HCV RNA levels showed the highest response rates, which matched the findings of other studies [10], [39]. Interestingly, but as reported previously, the favorable *IFNL3* rs12979860CC and rs8099917TT genotypes were strongly associated with high HCV RNA levels [12], [21]. Low HCV RNA levels independently predict SVR only in patients carrying unfavorable *IFNL3* genotypes.

Recently, a new polymorphism (ss469415590, ΔG/TT) was identified between the *IFNL2* and *IFNL3* genes which creates or disrupts an open reading frame in a new gene designated interferon lambda 4 (*IFNL4*) [30]–[32]. It was suggested that the presence of the functional interferon lambda 4 is associated with impaired HCV clearance and treatment response. However, in patients of European descent*IFNL4* provides comparable information because of its high linkage to the *IFNL3* rs12969860 SNP. Thus, the *IFNL4* is common in patients carrying the rs12979860 T-allele and up-regulates the expression of interferon-stimulated genes (ISG) before treatment. High pre-treatment intrahepatic ISG levels have been shown to be associated with poorer ISG response leading to reduced efficiency of HCV clearance [16], [24]–[28]. Since interferons alpha and lambda induce a large overlapping set of target ISGs, the genes are already activated at an intermediate level and their refractoriness to IFN alpha might be one mechanism responsible for non-response to IFN-based therapy observed in chronically infected patients. In vitro and in vivo studies demonstrated, that continuous exposure of hepatocytes to interferon results in reduced IFN sensitivity and the ISG expression maintains on pre-treatment level. Moreover, any further IFN treatment fails to re-induce transcription of ISGs [40]–[42]. However, further research is required to elucidate the relationship between the *IFLN3* genotypes and *IFLN4* and the impact on ISG expression affecting IFN-based treatment response.

We observed that patients exhibiting high cholesterol levels had an increased likelihood of achieving SVR compared to those with low levels, similar to previous reports [10], [11]. In contrast, low serum cholesterol concentrations correlated with non-response to IFN-based treatment [10], [11], hepatic steatosis [43], and more severe fibrosis [44]. Our study revealed a clear correlation between the homozygous *IFNL3* SNPs rs12979860CC, elevated cholesterol concentrations and a lower prevalence of steatosis, which is in line with previous reports [45]. This might be explained by the interaction between the lambda interferons and cholesterol metabolism on a cellular level. During interferon treatment, lipoprotein lipase is suppressed by increasing low density lipoprotein (LDL) cholesterol concentrations and decreasing triglyceride levels [46]. Cholesterol depletion may inhibit endocytosis of interferon lambda and suppress the activation of interferon lambda responsive cascades [47]. Carriers of the rs12979860CC genotype might be less exposed to such disturbances in lipid metabolism.

**Figure 4:**
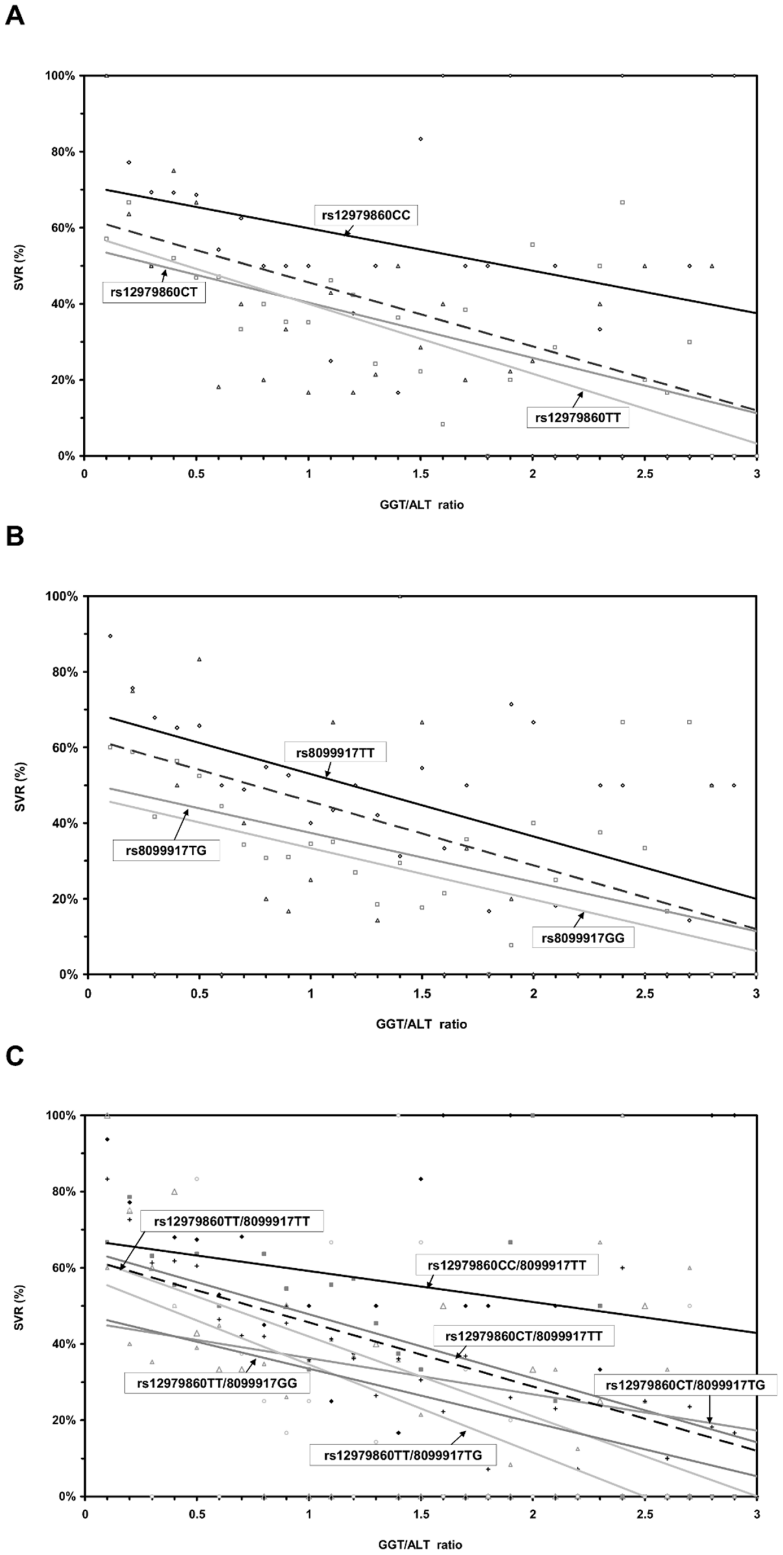
Impact of the GGT/ALT ratio on sustained virologic response (SVR) according to the *IFNL3* polymorphisms. GGT/ALT affects responsiveness of *IFNL3* (**A**) rs12979860 and (**B**) rs8099917 and the combination (**C**) rs12979860/rs8099917 in the overall cohort (n = 1402); depicted as single values with linear regression curves. The dashed line indicates the normal GGT/ALT ratio.

Although we still have no answer for the relationship between GGT levels and IFN responsiveness, high baseline GGT levels were found to be associated with non-responsiveness to interferon-based therapies in previous studies [7], [8]. We recently demonstrated [5] an inverse correlation between GGT and ALT, arguing that GGT elevations that were part of an ALT flare were less predictive for non-response than those GGT elevations that were seen in patients with low or even normal ALT levels. Indeed, a GGT to ALT ratio improved the specificity of response prediction [29]. We were able to confirm that this ratio was a better predictor compared to GGT alone. Furthermore, we identified associations of *IFNL3* rs12979860CC and rs8099917TT genotypes with low GGT values and high levels of ALT. Overall, the correlation between GGT levels and the rs8099917 SNP was less pronounced compared to the rs12979860 polymorphism. With increasing GGT levels, the SVR rates of carriers of at least one copy of the “non-responder” T- and G-allele were up to 3 times lower than those of patients possessing the “responder genotypes” rs12979860CC and rs8099917TT (Fig. 4). However, carriers of the homozygous responder *IFNL3* genotypes were similarly affected by the GGT/ALT ratio. In the presence of a high GGT/ALT ratio, carriers of the *IFNL3* rs12979860CC or rs8099917TT genotype showed reduced IFN responsiveness, similar to that observed in carriers of the non-responder alleles exhibiting low GGT/ALT ratios. As recently shown [37], the combined determination of both *IFNL3* SNPs provides more detailed information with respect to the likelihood of treatment response in patients carrying the heterozygous rs12979860CT genotype. Although the additional presence of the rs8099917TT allele increased the chance of achieving SVR, the pronounced effect of GGT and ALT activity on IFN responsiveness still remained (Fig. 4). As a consequence, besides *IFNL3* genotypes, the impact of GGT and ALT activity has to be considered and inclusion of these parameters into any decision algorithm seems to be beneficial for response prediction.

SVR rates significantly increased in HCV genotype 1 infection when protease-inhibitors-based therapies with a backbone of interferon and ribavirin entered the standard of care. Many factors involved in IFN responsiveness, such as *IFNL3* genotype [20], [23], [22], GGT, LDL [48], [49] and HCV RNA levels [50], still maintained their predictive potential. Furthermore, not only the *IFNL3* status, but also GGT levels play a role in some interferon-free direct-acting antiviral (DAA) regimens [51], highlighting the relevance of these markers in the mechanisms associated with the control of HCV infection. Therefore, the association of the *IFNL3* SNPs with certain biochemical parameters and their impact on treatment-induced clearance of infection might be of interest, independent from treatment strategies.

For correct interpretation of the results it has to be taken in account that the study has some limitations, because the cohort included patients of European descent with chronic HCV genotype 1 infection. Since the frequency of the *IFNL3* polymorphisms differs between ethnicities the improvement of response prediction by combining baseline parameters with the genetic variants might vary. Especially in patients with Afro-American ancestry the determination of *IFLN4* might be clearly more informative [30]–[32]. However, there is evidence that *IFNL4* might even affect HCV clearance and treatment prediction in patients of European descent [52]. Therefore, further research is required to elucidate the impact of *IFNL4* on the genetic association with biochemical predictors. Furthermore, since the impact of *IFLN3* SNPs on treatment response is lower in patients infected with HCV non-1 genotypes, the association of the polymorphism with baseline predictors might have different characteristics.

In conclusion, a clear correlation exists between the *IFNL3* genotype and the biochemical phenotype of patients of European descent infected with hepatitis C, including the levels of GGT, ALT, and cholesterol. These findings may explain the well-known predictive impact of certain biochemical markers on treatment outcome, and may provide new insights into the mechanisms by which innate immunity influences disease. Treatment outcome prediction can be improved by a combined determination of the *IFNL3* rs12979860 and rs8099917 polymorphisms and baseline predictors such as GGT, ALT and HCV RNA concentrations, thereby providing a better tool for decision making. Further work is required to elucidate the interplay of these parameters that appear to govern the outcome and the therapeutic response of patients with chronic HCV infection.

## Supporting Information

Figure S1 Association of *IFNL3* variants with baseline predictors in the replication cohort. Association of the *IFNL3* rs12979860 and rs8099917 genotypes with the levels of (**A)** GGT (IU/mL), **(B)** ALT (IU/mL), **(C)** GGT/ALT ratio, **(D)** pretreatment HCV RNA log_10_ concentration (IU/mL). Horizontal bars represent the median. Mann-Whitney U-test was used to compare the baseline parameter.
(TIF)

Table S1 Comparison of the convenience of GGT/ALT ratio, HCV RNA and *IFNL3* variants for response prediction in the evaluation and replication cohort.
(DOC)
