# Supplementary figures and images for: Association of IFNL3 rs12979860 and rs8099917 with Biochemical Predictors of Interferon Responsiveness in Chronic Hepatitis C Virus Infection
Source: PLoS One. 2013 Oct 29;8(10):e77530. doi: 10.1371/journal.pone.0077530 (PMC3812277; doi:10.1371/journal.pone.0077530)

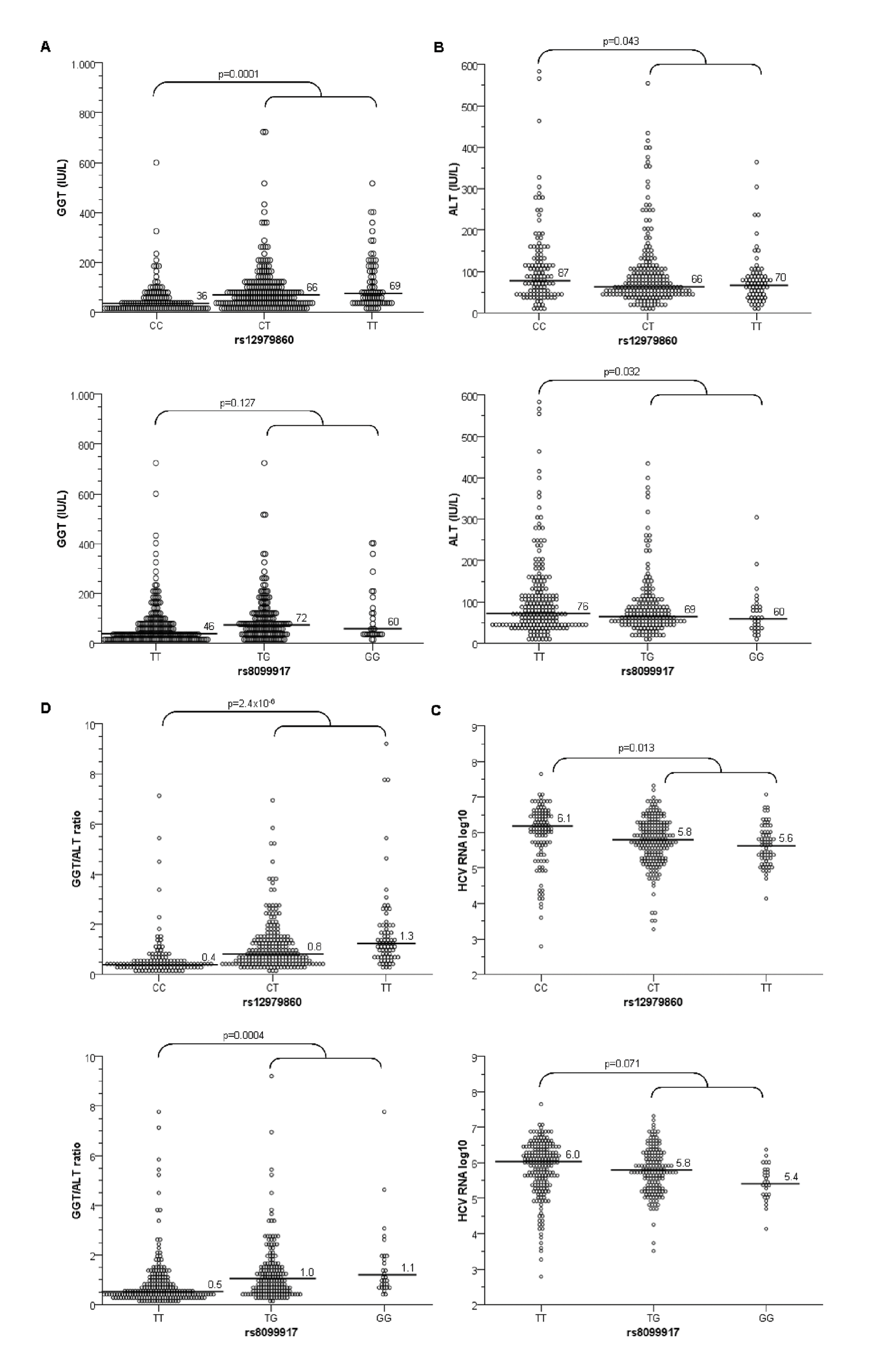

Supplement: Figure S1 — Association of IFNL3 variants with baseline predictors in the replication cohort. Association of the IFNL3 rs12979860 and rs8099917 genotypes with the levels of (A) GGT (IU/mL), (B) ALT (IU/mL), (C) GGT/ALT ratio, (D) pretreatment HCV RNA log10 concentration (IU/mL). Horizontal bars represent the median. Mann-Whitney U-test was used to compare the baseline parameter. (TIF) [file pone.0077530.s001.tif]
