# Supplementary material for: Association of IFNL3 rs12979860 and rs8099917 with Biochemical Predictors of Interferon Responsiveness in Chronic Hepatitis C Virus Infection
Source: PLoS One. 2013 Oct 29;8(10):e77530. doi: 10.1371/journal.pone.0077530 (PMC3812277; doi:10.1371/journal.pone.0077530)
Supplement: Table S1 — Comparison of the convenience of GGT/ALT ratio, HCV RNA and IFNL3 variants for response prediction in the evaluation and replication cohort. (DOC) [file pone.0077530.s002.doc]

Table S1: Comparison of the convenience of GGT/ALT ratio, HCV RNA and *IFNL3* variants for response prediction

|  | Evaluation cohort | | | | | Replication cohort | | | | |
| --- | --- | --- | --- | --- | --- | --- | --- | --- | --- | --- |
| Parameter | AUROC | Sensitivity | Specificity | PPV | NPV | AUROC | Sensitivity | Specificity | PPV | NPV |
| GGT/ALT ratio cut-off 0.70 | 0.705 | 63% | 67% | 61% | 68% | 0.666 | 63% | 61% | 54% | 70% |
| HCV RNA cut-off 5.8log_10_ (IU/mL) | 0.600 | 55% | 57% | 53% | 61% | 0.625 | 62% | 57% | 51% | 68% |
| rs12979860CC |  | 45% | 80% | 65% | 64% |  | 40% | 79% | 57% | 65% |
| rs8099917TT |  | 64% | 59% | 58% | 67% |  | 64% | 57% | 51% | 69% |

AUROC: area under receiver operating characteristics curve, PPV: positive predictive value, NPV: negative predictive value, GGT: gamma-glutamyltranspeptidase, IU: international units
